# The NBDC-DDBJ imputation server facilitates the use of controlled access reference panel datasets in Japan

**DOI:** 10.1038/s41439-022-00225-6

**Published:** 2022-12-20

**Authors:** Tsuyoshi Hachiya, Manabu Ishii, Yosuke Kawai, Seik-Soon Khor, Minae Kawashima, Licht Toyo-Oka, Nobutaka Mitsuhashi, Asami Fukuda, Yuichi Kodama, Takatomo Fujisawa, Katsushi Tokunaga, Toshihisa Takagi

**Affiliations:** 1 Genome Analytics Japan Inc., Tokyo, Japan; 2 Genome Medical Science Project, National Center for Global Health and Medicine, Tokyo, Japan; 3 Department of NBDC Program, Japan Science and Technology Agency, Tokyo, Japan; 4 Toyama University of International Studies, Toyama, Japan; 5 Database Center for Life Science, Chiba, Japan; 6 Bioinformation and DDBJ Center, National Institute of Genetics, Shizuoka, Japan

**Keywords:** Population genetics, Genetic association study

## Abstract

Accurate genotype imputation requires large-scale reference panel datasets. When conducting genotype imputation on the Japanese population, researchers can use such datasets under collaborative studies or controlled access conditions in public databases. We developed the NBDC-DDBJ imputation server, which securely provides users with a web user interface to execute genotype imputation on the server. Our benchmarking analysis showed that the accuracy of genotype imputation was improved by leveraging controlled access datasets to increase the number of haplotypes available for analysis compared to using publicly available reference panels such as the 1000 Genomes Project. The NBDC-DDBJ imputation server facilitates the use of controlled access datasets for accurate genotype imputation.

Genotype imputation is a crucial step in genome-wide association studies (GWASs). Polymorphic markers genotyped by DNA microarrays are called *tag single-nucleotide polymorphisms (SNPs)*. Genotype imputation infers the genotype of variants that are not directly observed in experiments but are in linkage disequilibrium (LD) with tag SNPs from the experimentally observed genotype of tag SNPs and *reference panels*, which are the collection of phased haplotypes.

Incorporating a large number of haplotypes in reference panels is important for accurate genotype imputation^1,2^. Although large-scale reference panel datasets^1,2^ are not publicly available, they can be made available for genotype imputation via *imputation servers*. Researchers can upload their datasets to the imputation server, configure parameters through a web user interface, execute genotype imputation on the server, and download the output files from the server.

In Japan, individual-level genetic data have been submitted to the Japanese Genotype-phenotype Archive (JGA)^3^, a database for *controlled access* datasets. Researchers need to apply for authorization to use controlled access datasets from the NBDC Data Access Committee. The NBDC security guidelines require that researchers store and process these controlled access datasets in secure data analysis environments and that data servers are owned by the data user or the organization to which data users belong or are *off-premise-server* described in the NBDC Guidelines for Human Data Sharing^4^. A reference panel dataset with >1000 Japanese ancestry subjects was submitted to the JGA^5^. In addition, whole-genome sequencing (WGS) data of ~2000 subjects of Japanese ancestry were recently registered in JGA^6^, and the number of WGS data will continue to increase in JGA. These datasets are important resources for achieving accurate genotype imputation for subjects of East Asian ancestry because reference panel datasets used in existing imputation servers, such as Michigan^7^ and TOPMed^1^, incorporate a smaller number of East Asians. Reference panel datasets from the GenomeAsia 100 K Project^8^, which are available at the Michigan imputation server, include >12,000 haplotypes of Asian ancestry across 64 countries, whereas those reference panel datasets place greater emphasis on South and Southeast Asians. Accordingly, East Asian-specific reference panels in JGA potentially have a different strength from the reference panels available at existing imputation servers. However, utilizing controlled access datasets in JGA for genotype imputation is difficult for researchers who do not have any data server compatible with the NBDC security guidelines or who require a user-friendly interface to execute genotype imputation workflows. To overcome these hurdles and to facilitate the use of controlled access reference panel datasets for genotype imputation, we developed a system called the NBDC-DDBJ imputation server.

The NBDC-DDBJ imputation server is composed of three modules (Fig. 1). The first is a web user interface that enables users to specify parameters. The second is a computational workflow that defines the inputs, steps, and outputs of genotype imputation. The third is the workflow execution system that performs the workflow.

**Fig. 1:**
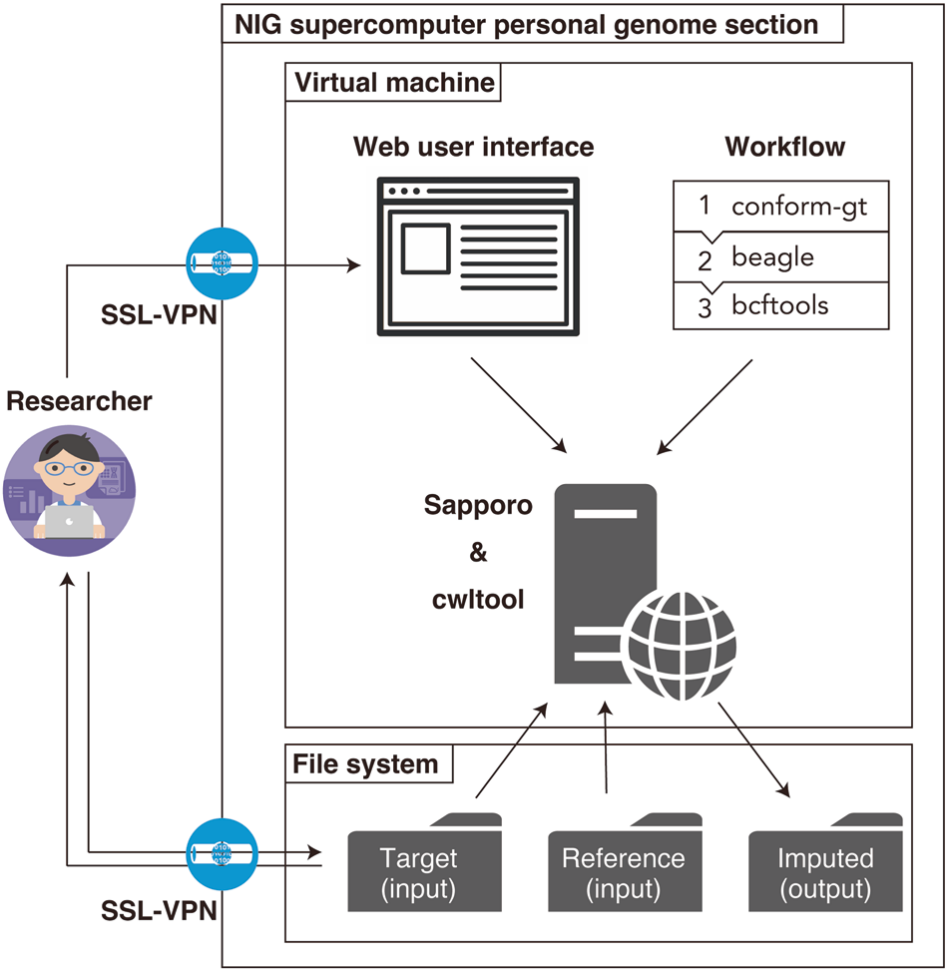
System overview of the NBDC-DDBJ imputation server. The NBDC-DDBJ imputation server is available in the personal genome analysis section of the NIG supercomputer. Researchers can upload their own genotype datasets to the server. They can access the web user interface to specify parameters and execute the workflow for genotype imputation. The workflow jobs are executed using Sapporo and cwltool in a virtual machine. The researchers can download the output files from the server. Through a secure socket layer virtual private network (SSL-VPN), users can securely use the system. By applying for authorization to use controlled access datasets from the NBDC Data Access Committee, if approved, users can access the datasets not only via the imputation server but also directly. NIG denotes the National Institute of Genetics; VPN virtual private network. The illustrations of the researchers are from TogoTV (©2016 DBCLS TogoTV/CC-BY-4.0).

## Workflow

We implemented the computational workflow using the Common Workflow Language (CWL)^9^. The workflow for the genotype imputation takes two genotype datasets as inputs. The first is an experimentally observed genotype dataset (referred to as the *target* dataset; let the number of subjects and variants be *n* and *m*, respectively), and the second is a reference panel genotype dataset (let the number of subjects and variants be *N* and *M*, respectively). Typically, *M* is substantially larger than *m* (*M* > *m*). Our workflow assumes that both genotype datasets are stored in the variant call format (VCF)^10^. We recommend that users apply quality control (QC) steps, such as missing call rate and Hardy-Weinberg equilibrium filters, to the target dataset before uploading to the imputation server (recommended criteria for QC steps are described elsewhere^11,12^). Regarding the nonpseudoautosomal region on the X-chromosome, our workflow assumes that male haploid genotypes are coded as “homozygous diploid” and that the male “homozygous diploid” and female heterozygous diploid genotypes are recoded in a single unphased VCF file according to an existing imputation workflow^13^. The first step of the workflow detects polymorphic markers shared between the two datasets using the conform-gt program (version 24May16)^14^, the second step performs prephasing and genotype imputation using the Beagle program version 5.2 (21Apr21.304)^15^, and the third step calculates the index for the imputed genotype files using bcftools (version 1.9)^16^. The workflow assumes that prephasing and genotype imputation were performed using the same reference panel dataset. These three steps can be executed in parallel by splitting genomic regions into chunks. The definition of chunks is configurable by editing a text file, whereas default configurations define a chunk for each whole chromosome. The output of the workflow includes the VCF files and their index files (TBI format). The VCF file contains the expected number of nonreference alleles (referred to as *allele dosage*) of *n* subjects and *M* variants in the DS tag. Estimated allele frequencies and imputation qualities^17^ are recorded in the INFO column of the VCF files. Our workflow can include not only autosomes but also the X-chromosome as the region for genotype imputation as long as the input reference panel dataset includes the phased genotype data of the X-chromosome. We are planning to extend our workflow to add options to choose software tools such as Eagle^18^ for prephasing and Minimac^7^ and IMPUTE^19^ for genotype imputation.

## Web user interface

**Fig. 2:**
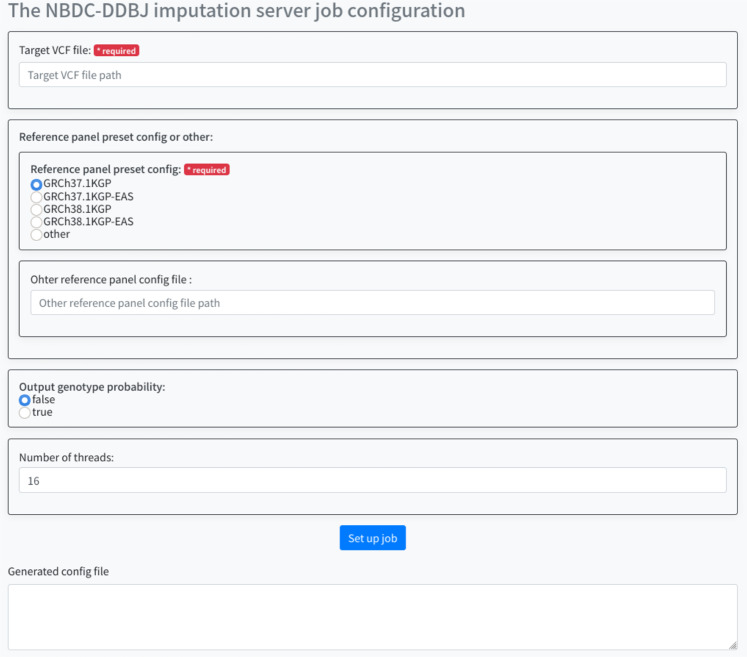
Web user interface of the NBDC-DDBJ imputation server. The mandatory parameters needed to execute the workflow for genotype imputation are (i) the file path to the target genotype dataset file and (ii) the reference panel configuration file that specifies chunks and the file paths to the reference panel genotype dataset and genetic map files. Optionally, users can specify the number of threads (default value of *16*) and whether the posterior probability of possible genotypes is included in the output file (default is *false*).

The mandatory parameters needed to execute the workflow for genotype imputation are (i) the file path to the target genotype dataset file and (ii) the reference panel configuration file that specifies chunks and the file paths to the reference panel genotype dataset and genetic map files (Fig. 2). Optionally, users can specify the number of threads (default value of *16*) and whether the posterior probability of possible genotypes is included in the output file (default is *false*). The web user interface enables users to specify these parameters graphically.

## Workflow execution system

The workflow for genotype imputation with specified parameters is executed using Sapporo^20^, an implementation of the workflow execution service (WES). We configured the Sapporo settings to use cwltool^21^ to parse and execute the workflow for genotype imputation.

## Reference panel datasets

We prepared six reference panel datasets for the NBDC-DDBJ imputation server (Table 1). Users can use reference panels provided by the 1000 Genomes Project (1KGP)^22,23^ without applying authorizations for data access. In addition, users can use a cross-imputed reference panel that combines the BioBank Japan (BBJ; *N* = 1037) and the 1KGP (*N* = 2504) datasets^5^. The cross-imputed reference panel was in the JGA under controlled access^5^. We extracted East Asian subjects from the reference panel datasets and constructed East Asian-specific reference panels. We converted the publicly available and controlled access reference panel datasets from the VCF to bref3 format using the bref3 program (version 28Jun21.220)^15^ to enable faster computation. The NBDC security guidelines do not distinguish the data access via imputation server from direct data access, and therefore, the users need to apply for authorization to use the reference panel datasets in JGA.

**Table 1:** List of the reference panel datasets.

Dataset name (human genome assembly)	Description	Data access level
1KGP_ALL (GRCh37)	A reference panel consisting of unrelated subjects of diverse ancestries (*N* = 2504) from the 1000 Genomes Project (phase 3, version 5). The X-chromosome is included.	Publicly available
1KGP_EAS (GRCh37)	A reference panel consisting of unrelated subjects of East Asian ancestries (*N* = 504) from the 1000 Genomes Project (phase 3, version 5). The X-chromosome is included.	Publicly available
1KGP_ALL (GRCh38)	A reference panel consisting of unrelated subjects of diverse ancestries (*N* = 2548) from the 1000 Genomes Project (30x on GRCh38). The X-chromosome is not included.	Publicly available
1KGP_EAS (GRCh38)	A reference panel consisting of unrelated subjects of East Asian ancestries (*N* = 508) from the 1000 Genomes Project (30x on GRCh38). The X-chromosome is not included.	Publicly available
BBJ1K + 1KGP_ALL (GRCh37)	A reference panel consisting of the BioBank Japan subjects of Japanese ancestry (*N* = 1037) and the 1000 Genomes Project (phase 3, version 5) subjects of diverse ancestries (*N* = 2504). In total, *N* = 3541. The X-chromosome is not included.	Controlled access
BBJ1K + 1KGP_EAS (GRCh37)	A reference panel consisting of the BioBank Japan subjects of Japanese ancestry (*N* = 1037) and the 1000 Genomes Project (phase 3, version 5) subjects of East Asian ancestries (*N* = 504). In total, *N* = 1541. The X-chromosome is not included.	Controlled access

1KGP denotes the 1000 Genome Project, *BBJ1K* the BioBank Japan subjects of Japanese ancestry (*N* = 1037), *EAS* East Asian.

## Accuracy

**Fig. 3:**
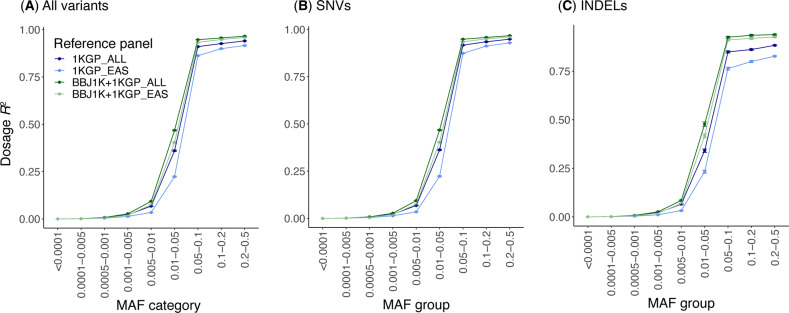
Comparison of imputation accuracy between reference panels. We randomly selected ten Japanese ancestry subjects from 1KGP, and their DNA microarray data were used as the target genotype dataset for benchmarking. The ten subjects were excluded from each reference panel dataset to avoid overestimation of imputation accuracy. Then, we performed the workflow for genotype imputation and evaluated the imputation accuracy (dosage *R*^2^) for various MAF categories. Variants on chromosome 22 were used for the benchmarking analysis. A single chunk was specified for the whole chromosome. Points and error bars indicate the mean and standard deviation of dosage *R*^2^, respectively. **A** Imputation accuracy of all variants. **B** Imputation accuracy of single-nucleotide variants (SNVs). **C** Imputation accuracy of insertions and deletions (INDELs). *1KGP* denotes the 1000 Genomes Project, *BBJ* Biobank Japan, *EAS* East Asian, *INDEL* insertion and deletion, *MAF* minor allele frequency, *SNV* single-nucleotide variant.

We evaluated the accuracy of the genotype imputation across various minor allele frequency (MAF) categories using four reference panels based on GRCh37 (Fig. 3A). The results showed that East Asian-specific reference panels were less accurate than the corresponding reference panels, including subjects of diverse ancestries. The cross-imputed reference panels of BBJ and 1KGP achieved higher accuracy than the 1KGP reference panels. The superior accuracy of the cross-imputed reference panels over the 1KGP reference panels was more evident in insertions and deletions (INDELs) than in single-nucleotide variants (SNVs) (Fig. 3B, C). Taken together, the use of the cross-imputed panel of diverse ancestries is recommended for accurate genotype imputation.

## Speed

The wall-clock time of executing genotype imputation (*n* = 2318) with 16 threads was 56.2 and 91.3 h when using the cross-imputed panels of East Asian-specific and diverse ancestries, respectively.

## Portability

The NBDC-DDBJ imputation server is currently available in the personal genome analysis section of the National Institute of Genetics (NIG) supercomputer. The system is highly portable because the workflow is fully containerized and implemented in CWL. Thus, the NBDC-DDBJ imputation server can be installed in other secure data analysis environments, including on-premise and cloud servers.

In summary, we developed the NBDC-DDBJ imputation server to provide users with a graphical user interface to perform genotype imputation within a secure data analysis environment. We also prepared ready-to-use reference panels, enabling users to use them without the need for intensive computation of whole-genome sequencing data analysis. The number of whole-genome sequencing data has been rapidly growing in the JGA; therefore, we will continue to construct and provide larger-scale reference panel datasets. The NBDC-DDBJ imputation server and ready-to-use reference panels will facilitate the use of controlled access datasets and contribute to improved genotype imputation accuracy.

## Software availability

The source code for the genotype imputation workflow is available at https://github.com/ddbj/imputation-server-wf. A container image for executing the workflow is available at https://github.com/orgs/ddbj/packages/container/package/beagle-5.2. The source code of the web user interface is available at https://github.com/ddbj/imputation-server-ui. These source codes are publicly available under Apache License 2.0. The NBDC-DDBJ imputation server system is available in the personal genome analysis section of the NIG supercomputer, which is available not only in Japan but also worldwide as long as the conditions of login users are met (https://sc.ddbj.nig.ac.jp/en/application/). The steps to apply for an NIG supercomputer account are described at https://sc.ddbj.nig.ac.jp/en/personal_genome_division/pg_application/. The steps to apply for authorization to access the controlled access datasets are described at https://humandbs.biosciencedbc.jp/en/data-use. Once the application is approved, the controlled access datasets can be used in Japan and abroad and by academic and commercial users. The cross-imputed reference panel for genotype imputation is accessible through the JGA upon request (accession code: JGAD000679).
